# Parathyroid adenoma apoplexy as a temporary solution of primary hyperparathyroidism: a case report

**DOI:** 10.1186/1752-1947-1-139

**Published:** 2007-11-17

**Authors:** Francisco A Pereira, Daniel F Brandão, Jorge Elias, Francisco JA Paula

**Affiliations:** 1 Department of Internal Medicine, School of Medicine of Ribeirão Preto, University of São Paulo, Ribeirão Preto, Brazil

## Abstract

**Introduction:**

The natural history of patients with spontaneous parathyroid necrosis is unknown. In this case report we describe the clinical course, laboratory, radiographic, bone densitometry tests, parathyroid ultrasonography and scintigraphy examinations of a patient performed over a period of eight years after she first presented with a sudden episode of spontaneous resolution of primary hyperparathyroidism (PHPT).

**Case presentation:**

A 24-year-old woman with a clinical history and laboratory and radiographic tests compatible with PHPT suffered a sudden episode of cervical pain and presented with clinical evidence of hypocalcemia. Biopsy of a cervical nodule revealed necrotic material compatible with ischemia of the parathyroid. The follow-up of the patient presented four distinct phases: the first, which lasted two years, was compatible with a period of bone hunger during which it was necessary to introduce calcitriol and calcium carbonate. During this period, the patient showed bone mass gain. The second phase was characterized by normalization of calcium and parathyroid hormone levels and its end was difficult to define. During the third phase there was a recurrence of hypercalcemia associated with elevated parathyroid hormone (PTH) levels and loss of bone mass. The last phase corresponded to the interval after parathyroidectomy, which was characterized by normalization of serum levels of calcium and PTH, as well as bone mass gain.

**Conclusion:**

This case report indicates that spontaneous resolution of PHPT by adenoma necrosis is potentially temporary. Thus, in cases in which a conservative approach is chosen, clinical and laboratory follow-up is indispensable. Bone mass measurement is a useful tool in the follow-up of these cases. However, this option exposes the patient to a potential roller-coaster ride of bone mass gain and loss, whose long term consequences are still unknown.

## Introduction

Although the natural history of primary hyperparathyroidism (PHPT) is incompletely understood, in many cases the calcium level remains stable when monitored on a regular basis [1,2]. In parallel, over the last two decades the number of reported cases that do not follow this pattern has increased [3]. The first case of cyclic PHPT was recently reported [4] and sporadic cases of spontaneous resolution due to apoplexy of parathyroid adenomas have been reported more frequently [3,5,6]. In the latter situation most patients were submitted to early surgery or were followed for as long as 30 months but this did not include bone mass evaluation.

In this case study we report the details of a patient who presented with spontaneous remission of PHPT and was followed for eight years after the apoplexy episode, at which time she presented with recurrence of hypercalcemia. In this case, in addition to obtaining clinical and laboratory documentation, the authors prospectively evaluated the patient's bone densitometry.

## Case presentation

**Figure 1:**
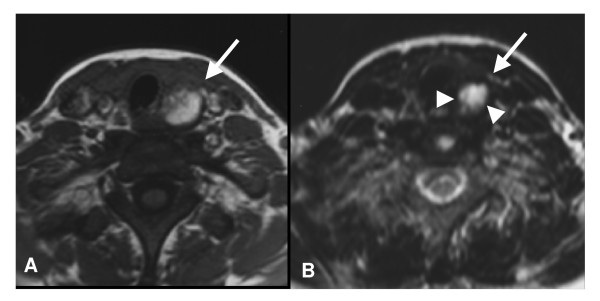
**Left inferior parathyroid adenoma**. Axial MR imaging scans (07/1998) weighted in T1 (A) and T2 (B) show a spontaneous hyperintense and well defined round lesion in both T1- and T2-weighted in the left-inferior parathyroid adenoma region (arrow). There is a hypointense lesion with a halo in the T2-weighted image which can represent degradation products of hemoglobin (arrowheads).

In 1998, a 24-year-old white female attended the endocrinology outpatient clinic of the Medical Center of the School of Medicine of Ribeirão Preto, USP, with complaints of bone pain and muscle weakness of ten years duration. She reported pain intensification in the upper and lower limbs for the past two years. In addition, over the past year she had started to develop nocturia, asthenia, and weight loss of about 8 kg. She also had had episodes of renal colic. She reported that long bone radiography had shown evidence of bone lesions. According to the patient, a bone biopsy had revealed the presence of a benign tumor. When seen at our outpatient clinic, the patient was found to be in good general condition, without any detectable cervical nodule. The patient had already had the following tests: calcium = 11.9 mg/dl, phosphorus (Pi) = 1.8 mg/dl, and alkaline phosphatase (ALP) = 1642 U/L. The initial by in our service confirmed the previous biochemical profile: calcium = 11.9 mg/dl (8.5–10.5 mg/dl), phosphorus = 1.7 mg/dl (2.5–4.5 mg/dl), ALP = 2352 U/L (40–160 U/L), creatinine = 0.5 mg/dl (0.7–1.1 mg/dl) chloride = 106 mEq/L (98–106 mEq/L), ionized calcium Ca^++ ^= 1.57 mmol/L (1.14–1.29 mmol/L), and chloride/Pi ratio = 62.3 (<30). Radiotransparent circular images with well defined margins without peripheral sclerosis were observed in the middle diaphyseal third of the left femur and left humerus and in the proximal diaphyseal third of the right tibia. Hand and skull radiographies only revealed osteopenia. One week after our first evaluation, she started to experience paresthesia in the hands, face and limbs and she experienced an episode of hand muscle contraction. Because of the persistence of paresthesia, the patient returned to our service, when Chvostek's sign was observed. Upon clinical examination, a painless nodule was palpated in the left lobe of the thyroid. Table 1 shows the laboratory evaluation. Cervical ultrasound revealed an enlarged left lobe of the thyroid with a nodular image measuring 7.4 cm^3^, with a heterogeneous echotexture and containing areas of cystic degeneration. Scintigraphic examination of the parathyroid with sestamibi did not show abnormal uptake. Magnetic resonance examination revealed a nodule (1.9 cm) with a cystic component with defined contours, compatible with a lesion of the parathyroid (Figure 1). Cytological examination showed a large amount of cell debris, few macrophages and neutrophils and numerous clusters of small anucleated necrotic cells or cells rarely containing pyknotic nuclei.

**Table 1:** Laboratory work-up upon patient admission

	Results (reference)
Calcium (mg/dl)	6.8 (8.5–10.5)
Ionized calcium (mmol/L)	0.95 (1.14–1.29)
PTH (pg/ml)	110 (6–67)
T_4_L (ng/dl)	0.8 (0.7 – 1.7)
TSH (UI/ml)	1.5 (0.4 – 4.0)
ALP (U/L)	847 (40–160)
Chloride (mEq/L)	106 (98–107)
Magnesium (mEq/L)	2.0 (1.6–2.6)
Creatinine (mg/dl)	0.4 (0.7–1.0)
LH (mUI/ml)	2.0 (0.3 – 13)
FSH (mUI/ml)	14.0 (13 – 30)
Prolactin (ng/dl)	14.0 (<25.0)
Plasma cortisol (μg/dl)	6.4 (5.0 – 20.0)
25-Hydroxyvitamin D (ng/ml)	11.0 (15.0 – 80.0)
1,25(OH)_2_D (pg/ml)	210 (15.0 – 60.0)

**Figure 2:**
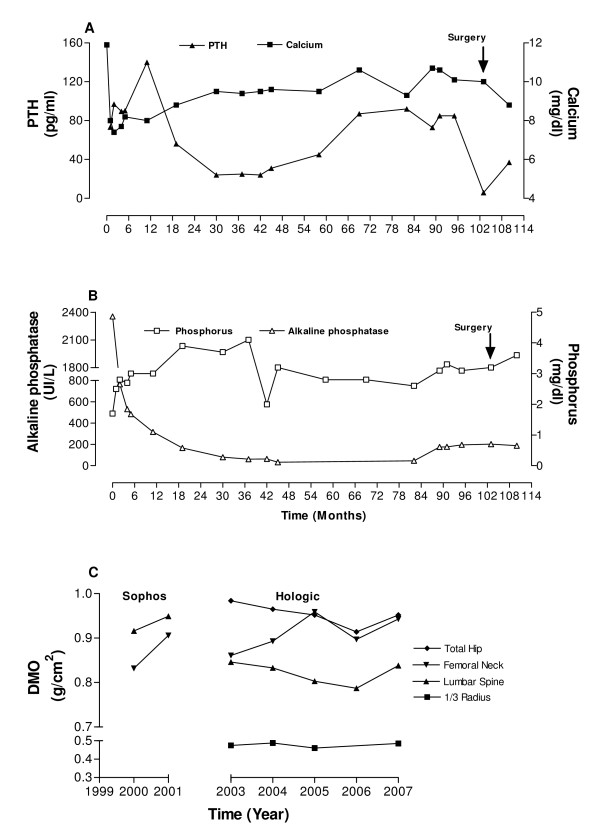
**Clinical investigation**. Evolution of serum levels of (A) PTH and calcium; (B) alkaline phosphatase and phosphorus and (C) bone mineral density of total hip, femoral neck, lumbar spine and distal third of radius (2000–2001, Sophos and 2003–2007 Hologic 4500 W equipment) during patient follow-up.

It was decided to use a noninvasive approach, using 1000 mg elemental calcium and 0.5 μg calcitriol/day (Roche, São Paulo, SP, Brazil). The neuromuscular symptoms of the patient were relieved and she continued to use this medication for 3 years. During this period her total calcium levels were always normal (8.0 to 10.4 mg/dl), although her Ca^++ ^levels were discretely low for 2 years (1.0 to 1.13 mmol/L) and then normal, during the third year (1.2 to 1.24 mmol/L). Pi levels were always normal (3.0 to 4.1 mg/dl) and PTH level was high during the first year (89.5 to 159 pg/ml) and normal during the second and third years (24–56 pg/ml). ALP levels progressively fell, ranging from 781 down to 317 U/L during the first year, from 266 to 94 U/L during the second year, and from 80 to 57 U/L during the third year. Calciuria was low throughout the three-year period (12.9 to 75 mg/24 hours). During the fourth year, calcitriol was first discontinued, soon followed by discontinuation of calcium as the patient started to present calcium levels in the upper normal limit. Between 2002 and 2004, the patient presented fluctuation of total calcium levels from 9.1 to 10.6 mg/dl, of Ca^++ ^from 1.21 to 1.44 mmol/L, of Pi from 2.0 to 3.5 mg/dl, of calciuria from 78,2 to111 mg/24 hours, and of PTH from 31 to 87 pg/ml. In 2004, 25-hydroxyvitamin D level was 19 ng/ml. In 2005 the patient showed a continuous tendency to elevation of calcemia, and parathyroidectomy was indicated. However, the patient agreed to submit to the procedure only in the middle of 2006. Figure 2A &2B shows the variation in laboratory measurements between 2005 and 2006.

Parathyroid scintigraphy with sestamibi in 2003 and before surgery did not show abnormal uptake of the radiopharmaceutical. Ultrasound showed a reduction of the nodule only in 2006, when its size was 0.53 cm^3^.

Bone mineral densitometry (BMD) was obtained using two different instruments. In 2000 and 2001, the patient showed bone mass gain in lumbar spine (3.3%) and femoral neck (7.4%), (Figure 2C). These examinations were performed with a Sophos L-XRA instrument (Sophos, Paris, France), which did not include software for the forearm. Starting in 2003, the examinations were performed with Hologic instruments (4500 W, Waltham, MA, USA, QDR System Software Version 11.2). In 2003 and 2006 BMD was: a) L1–L4 = 0.846 and 0.787 g/cm^2^; b) total hip = 0.984 and 0.914 g/cm^2 ^and c) 1/3 distal radius = 0.475 and 0.423, respectively (Figure 2C).

The preoperative results obtained for the patient were as follows: total calcium= 10.7 mg/dl, Ca^++ ^= 1.38 mmol/L, phosphorus = 3.1 mg/dl, ALP = 186 U/L, and PTH = 73 pg/ml.

**Figure 3:**
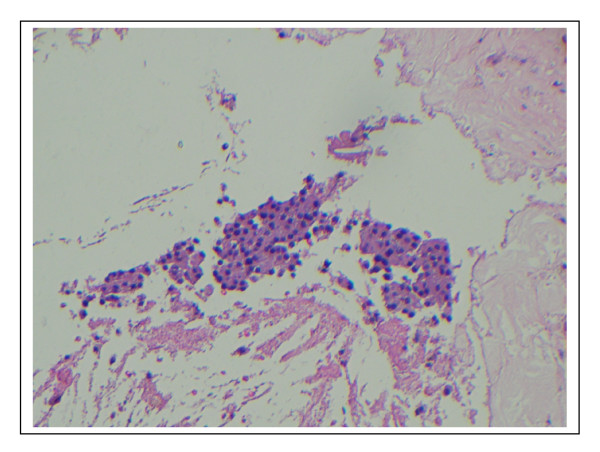
**Photomicrograph of the parathyroid adenoma**. Image showing extensive areas of necrosis represented by eosinophilic amorphous material, with fibrosis, and a few remaining clusters of tumoral cells (Hematoxylin-Eosin, ×100).

The patient underwent conventional surgery with bilateral neck exploration under general anesthesia in July 2006, when an adenoma was located in the left inferior pole of the thyroid. A blood sample was collected 10 minutes after the resection of the suspected hyperfunctioning parathyroid tissue for later determination of serum PTH by a conventional method. The result (PTH = 5.9 pg/ml) supported the success of the surgical procedure. Anatomopathological examination showed a benign proliferation of parathyroid cells, the presence of fibrosed tissue with hemosiderin deposition (old hemorrhage), and a cavity surrounded by fibrous walls containing macrophages, lymphocytes, multinucleated giant cells, cholesterol crystals and cell debris (Figure 3). Postoperatively the patient showed normalization of serum total calcium levels (8.9 mg/dl), Ca^++ ^(1.15 mmol/L), phosphorus (3.1 mg/dl), albumin (4.3 g/dl) and ALP (165.0 U/L). In addition, we observed significant increase in BMD in all sites evaluated six months after parathyroidectomy (L1–L4 6.5%, 1/3 of radius 5.4%, femoral neck 5.1% and total hip 4.1%), Figure 2C.

## Discussion

In view of the current status of primary hyperparathyroidism as a frequent disease that is usually asymptomatic, a challenge regarding the management of patients with this disease is to identify the best treatment option, considering that surgery is the only curative option [7].

After almost two decades of surveillance of patients in a chronic PHPT state, it is necessary to point out other aspects that used to be imperceptible and/or rare and that today represent new challenges regarding the treatment of these patients. Patients with PHPT presenting with normal serum PTH levels have been occasionally reported [8]. However, because of its frequency, the occurrence of spontaneous PHPT resolution by apoplexy of parathyroid adenomas is even more important. Onoda et al. [9] in 1994 stated that only 25 such cases had been reported. However, the frequency of this problem has increased and at least 26 additional similar cases have been reported since that time [3]. These patients either submitted to surgery early [5,6], or were followed for about 3 years, and there is no information about a natural evolution monitored over a more prolonged time with ultrasonographic and scintigraphic evaluation. In particular, none of these studies reported the evolution of BMD in these patients. In this case report we describe the evolution of all of these parameters over a period of 8 years in a patient who presented with spontaneous remission of PHPT followed by recurrence of the functional disorder at the end of this 8 year period. Based on this case, it is possible to extract important information regarding appropriate clinical and surgical conduct and the most appropriate timing for surgery.

The first aspect to be emphasized in this case is that punch biopsy of the parathyroid, at the time of adenoma infarction, associated with anatomopathologic examination after parathyroidectomy of the same gland, showed evidence of previous necrosis and benign proliferation of parathyroid cells indicating recurrence of parathyroid adenoma.

The first phase, which followed the infarction of the parathyroid adenoma, is compatible with a state of bone hunger, which is expected in patients with the important bone disorder of PHPT, as demonstrated in the present case by the elevation of alkaline phosphatase and the clearly visible radiologic changes of PHPT. During this period PTH remained elevated as a physiological adaptation to the increased calcium utilization by bone and bone mass recomposition. However, the periods of apparent normalization of parathyroid functioning that follow this phase and the later recrudescence of PHPT are difficult to delimit. This is largely due to the fluctuation of serum calcium and PTH levels that may occur in these patients. The undefined signs and symptoms may produce an undesirable loss of bone mass and non-quantifiable changes in bone quality that may not be fully corrected after definitive surgery. This aspect should be particularly considered in order to define the most appropriate therapeutic course. In this reported case, measurement of bone mass proved to be a sensitive and objective procedure for the demonstration of disease activity and was useful regarding the decision about the need for surgery.

Currently, different centers are performing parathyroid surgery by minimally invasive procedures (MIP) with huge success, thus providing a less traumatic and attractive alternative therapy for PHPT [10,11]. The standardization of these procedures and of the examinations that usually are associated with these procedures, such as optimizing parameters of parathyroid localization (intraoperative scintigraphic scan) and to assure surgical success (intraoperative determination of quick PTH) will allow the use of these therapeutic options on a large scale [12]. However, in relation to the patient described herein there are some concerns about doing a MIP: a) the patient had a negative scan with sestamibi, b) the possible presence of more than one parathyroid nodule and c) the previous parathyroid apoplexy could result in morphological alterations secondary to hemorrhage. It was considered that this patient should be included in the group for which conventional surgery would be more appropriate. According to Micolli et al., (2000) [10] approximately 30% of the patients with PHPT are suitable for conventional surgery.

## Conclusion

This case indicates that the apparent cure that follows necrosis of a parathyroid adenoma is potentially temporary. All data suggest that an early surgical approach is acceptable for these patients. Conservative management, which involves periodic clinical and laboratory examinations, definitely results in higher costs. Our data also suggest that in addition to calcium and PTH measurements, bone densitometry is a most useful tool for the follow up of these patients.

## Competing interests

The author(s) declare that they have no competing interests.

## Authors' contributions

FAP and FJAP were responsible for the initial evaluation and management of the patient. DFB was responsible for anatomopathologic examinations. JEJ performed the radiological analysis. FAP, DFB, JEJ and FJAP critically revised the content of the manuscript. All authors have read and approved the final version of the manuscript.

## Consent

Written informed consent was obtained from the patient for publication of this case report.

## References

[B1] Consensus development conference panel. diagnosis and management of asymptomatic hyperparathyroidism: consensus development statement Ann Intern Med 1991 114 593 597 1900404 10.7326/0003-4819-114-7-593

[B2] Nylen E Shah A Hall J Spontaneous remission of primary hyperparathyroidism from parathyroid apoplexy J Clin Endocrinol Metab 1996 81 1326 1328 10.1210/jc.81.4.1326 8636326

[B3] Wootten CT Orzeck EA Spontaneous remission of primary hyperparathyroidism: a case report and meta-analysis of the literature Head Neck 2006 28 81 88 10.1002/hed.20316 16284975

[B4] Makita N Iiri T Sato J Fukumoto S Okazaki T Yamazaki K Obara T Fujita T An instructive case suggesting cyclical primary hyperparathyroidism Endocr J 2006 53 311 316 10.1507/endocrj.K05-097 16710075

[B5] Lucas DG Jr Lockett MA Cole DJ Spontaneous infarction of parathyroid adenoma: two cases report and review of literature Am Surg 2002 68 173 176 11842966

[B6] Kovacs KA Gay JD Remission of primary hyperparathyroidism due to spontaneous infarction of a parathyroid adenoma. Case report and review of the literature Medicine 1998 77 398 402 10.1097/00005792-199811000-00006 9854603

[B7] Bilezikian JP Potts JT Jr Fuleihan Gel-H Kleerekoper M Neer R Peacock M Rastad J Silverberg SJ Undelsman J Wells SA Jr Summary statement from a workshop on asymptomatic primary hyperparathyroidism: a perspective for the 21^st ^century J Bone Miener Res 2002 17 2 11 12412771

[B8] Mischis-Troussard C Goudet P Verges B Cougard P Tavernier C Maillefert JF Primary hyperthyroidism with normal serum intact parathyroid hormone levels QJM 2000 93 365 367 10.1093/qjmed/93.6.365 10873186

[B9] Onoda N Miyakawa M Sato K Demura H Uchida E Spontaneous remission of parathyroid adenoma followed with ultrasonographic examinations J Clin Ultrasound 1994 22 134 136 10.1002/jcu.1870220213 8132794

[B10] Miccoli P Berti P Conte M Raffaelli M Materazzi G Minimally invasive video-assisted parathyroidectomy: lesson learned from 137 cases J Am Coll Surg 2000 191 613 618 10.1016/S1072-7515(00)00737-7 11129809

[B11] Politz D Livinqston CD Victor B Askew R Jones L Minimally invasive radio-guided Parathyroidectomy in 152 consecutive patients with primary hyperparathyroidism Endocr Pract 2006 12 630 634 17229658 10.4158/EP.12.6.630

[B12] Vignali E Picone A Materazzi G Steffe S Berti P Cianferotti L Cetani F Ambrogini E Miccoli P Pinchera A Marcocci C A quick intraoperative parathyroid hormone assay in the surgical management of patients with primary hyperparathyroidism: a study of 206 consecutive cases Eur J Endocrinol 2002 146 783 788 10.1530/eje.0.1460783 12039698

